# Targeted Lipidomics and Lipid Metabolism Elucidate Anti-Obesity Effects of Lactic Acid Bacteria-Fermented Purple Sweet Potato Tainung No. 73 Extract in Obese Mice

**DOI:** 10.3390/ijms27031489

**Published:** 2026-02-03

**Authors:** Hsien-Yi Yang, Chien-Hsun Huang, Shang-Tse Ho, Hsin-Hui Su, Yen-Po Chen, Yung-Tsung Chen

**Affiliations:** 1 Department of Food Science, National Taiwan Ocean University, Keelung 202301, Taiwan; eagle871009@gmail.com (H.-Y.Y.); shwdi2119@gmail.com (H.-H.S.); 2 Bioresource Collection and Research Center (BCRC), Food Industry Research and Development Institute, Hsinchu 30062, Taiwan; chh@firdi.org.tw; 3 Department of Wood-Based Materials and Design, National Chiayi University, Chiayi 600355, Taiwan; stho@mail.ncyu.edu.tw; 4 Department of Animal Science, National Chung Hsing University, Taichung 40227, Taiwan; chenyp@dragon.nchu.edu.tw; 5 The iEGG and Animal Biotechnology Research Center, National Chung Hsing University, Taichung 40227, Taiwan

**Keywords:** purple sweet potato, fermentation, lipidomics, lipid metabolism, obesity

## Abstract

The increasing prevalence of obesity and metabolic disorders poses a major global health challenge. In the present study, purple sweet potato Tainung No. 73 was fermented using *Lactobacillus amylovorus* OFMLa-73 and *Levilactobacillus brevis* OFMLb-143 to enrich the specific bioactive metabolite indolelactic acid. Furthermore, supplementation with fermented sweet potato (FSPE) ethanol extract resulted in a significant reduction in body weight gain, adipocyte hypertrophy, and hepatic lipid accumulation, while also improving serum lipid profiles in high-fat diet-induced obesity mice. These physiological improvements were associated with the downregulated expression of adipogenic and inflammatory genes in both liver and adipose tissues. Furthermore, lipidomic analysis revealed that FSPE modulated key lipid species, including ceramides and acylcarnitines, which are implicated in metabolic dysfunction. Collectively, these findings demonstrated that lactic acid fermentation enhanced purple sweet potato’s functional potential, positioning FSPE as a promising candidate for dietary intervention in obesity management.

## 1. Introduction

The global prevalence of obesity and metabolic disorders is rising at an alarming rate, posing serious public health challenges. Obesity is a major contributor to various metabolic diseases and is frequently associated with metabolic dysfunction-associated steatotic liver disease (MASLD). Conventional anti-obesity treatments often demonstrate limited efficacy and are accompanied by adverse side effects, reducing their overall therapeutic value [1]. Consequently, there is a growing interest in developing natural bioactive compounds as alternative treatment strategies.

Sweet potato, a nutrient-dense food rich in dietary fiber and bioactive compounds, has garnered increasing attention for its potential health benefits. It contains high levels of antioxidants, including polyphenols and flavonoids such as chlorogenic acid [2], as well as anthocyanins known for their health-promoting effects [3]. A range of studies have indicated that sweet potato extracts exhibit hepatoprotective [4], anti-obesity [5], anti-inflammatory [6], antioxidant [7], and prebiotic [8,9] properties, underscoring their potential as a functional food ingredient for health enhancement.

In recent years, fermentation has emerged as an effective strategy to enhance the nutritional profile and bioactivity of plant-based foods. Notably, fermentation of sweet potato with lactic acid bacteria (LAB) has been shown to improve the bioavailability and stability of bioactive compounds, thereby amplifying its health-promoting potential [10,11]. Previous studies have demonstrated that fermented plant-based foods can increase the level of specific bioactive compounds, reduce lipid synthesis, and enhance energy metabolism in both in vitro and in vivo models [12,13].

Lipidomics is a powerful approach for investigating lipid metabolism in the context of metabolic disorders, particularly obesity. Analyzing changes in serum metabolite profiles can help elucidate the roles of various lipid species in disease progression [14]. Obesity significantly alters lipid profiles, particularly affecting phosphatidylcholines, phosphatidylethanolamines, and lysophosphatidylcholines [15,16]. Additionally, metabolic pathways involving glycerophospholipids, α-linolenic acid [17], and linoleic acid [18] are closely associated with obesity-related lipid dysregulation.

Although fermentation is known to enhance the bioavailability and stability of bioactive compounds, the specific metabolites responsible for its anti-obesity effects remain poorly characterized. Furthermore, the molecular mechanisms underlying the effects of fermented sweet potato treatment on obesity remain poorly elucidated. Therefore, a comprehensive investigation into lipid modulation following fermented sweet potato intervention is essential to gain scientific insight into its potential role in obesity management.

This study aimed to evaluate the levels of total phenolics, flavonoids, and anthocyanins in purple sweet potato Tainung No. 73 fermented with selected LAB strains. Additionally, ethanol-extracted metabolites were characterized, and their functional effects on lipid metabolism were assessed using an obese mouse model. By integrating fermentation technology with lipidomics, this study aimed to elucidate the molecular pathways underlying the anti-obesity potential of fermented sweet potato, thereby offering a promising dietary strategy to improve metabolic health.

## 2. Results

### 2.1. Fermentation Screening and Extraction Efficiency

**Figure 1:**
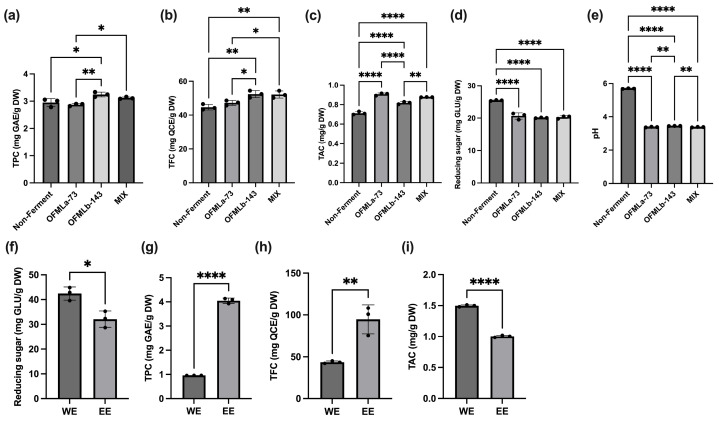
Effects of lactic acid bacteria fermentation on physicochemical properties and bioactive compounds of purple sweet potato. (**a**) Total phenolic content (TPC); (**b**) total flavonoid content (TFC); (**c**) total anthocyanin content (TAC); (**d**) reducing sugar content in water extracts (WE) and ethanol extracts (EE); (**e**) pH value of fermented samples; (**f**) reducing sugar content; (**g**) TPC; (**h**) TFC; and (**i**) TAC of water extracts and ethanol extracts. Data are presented as mean ± standard deviation (SD), based on biological replicate samples (*n* = 3). Statistical analysis was performed using one-way analysis of variance (ANOVA) followed by Tukey’s post hoc test. Statistical significance is indicated as follows: * *p* < 0.05, ** *p* < 0.01, and **** *p* < 0.0001.

To evaluate the impact of LAB-based fermentation, the contents of total phenolics, flavonoids, and anthocyanins were quantified. The test strains were selected from five candidate strains (*L. amylovorus* OFMLa-73, *Pediococcus pentosaceus* OFMPp-107, *Lactiplantibacillus plantarum* OFMLp-141, *L. brevis* OFMLb-143-1, and *L. brevis* OFMLb-143) from laboratory collection based on a preliminary screening that evaluated their ability to enhance the content of bioactive compounds in water extract (Figure S1). The results indicated that *L. amylovorus* OFMLa-73 exhibited the highest efficiency in increasing anthocyanin content, while *L. brevis* OFMLb-143 was the most effective strain for enhancing total phenolic and flavonoid levels among the tested strains. Consequently, these two strains were selected for subsequent experiments. Among the tested strains, *Lactobacillus amylovorus* OFMLa-73 significantly enhanced the anthocyanin concentration, whereas *Levilactobacillus brevis* OFMLb-143 markedly increased total phenolic and flavonoid levels (Figure 1a–c) in ethanol extract. Notably, co-fermentation using a mixed-strain consortium further elevated total flavonoid and anthocyanin contents in ethanol extract compared to non-fermented purple sweet potatoes.

A substantial reduction in reducing sugar levels was observed across all fermented groups, with OFMLb-143 achieving the most significant decrease (Figure 1d). The decline in pH values post-fermentation indicated robust microbial acidification, confirming the effective fermentation of LAB strains at 37 °C for 48 h (Figure 1e). This acid production aligns with the enhanced bioactivity observed in the fermented products.

To optimize the extraction of bioactive compounds, co-fermented sweet potato samples were processed using either hot water or 70% ethanol. Ethanol extraction significantly reduced residual reducing sugars by approximately 25%, while yielding a 4-fold increase in total phenolic content and a 2-fold increase in total flavonoid content (Figure 1f–h). In contrast, total anthocyanin content was approximately 33% lower in the ethanol extract compared to the hot water extract, indicating superior preservation of anthocyanins via aqueous extraction (Figure 1i). Collectively, these findings highlighted ethanol as the more effective solvent for concentrating polyphenols and flavonoids. Consequently, the FSPE obtained via ethanol extraction was selected for subsequent in vivo analysis.

### 2.2. FSPE Reduces Body Weight Gain and Adiposity in High-Fat Diet-Induced Obese Mice

**Figure 2:**
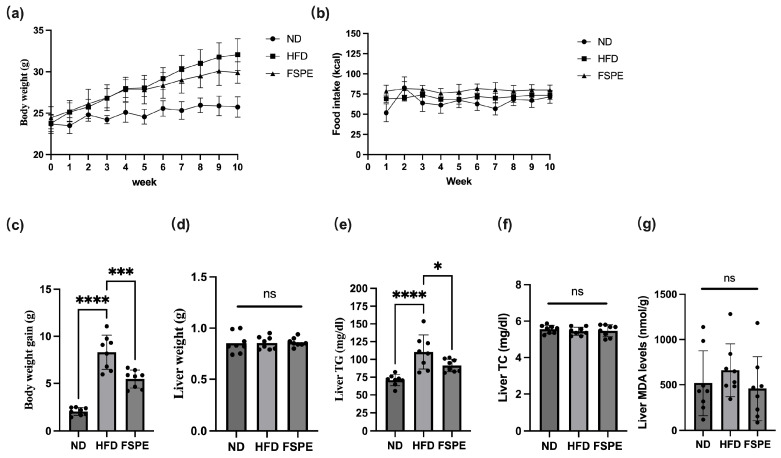
Effect of FSPE supplementation on body weight, weight gain, liver weight, and hepatic lipid droplet characteristics in high-fat diet (HFD)-fed mice. (**a**) Body weight, (**b**) food intake. (**c**) body weight gain, (**d**) liver weight, (**e**) hepatic triglyceride content, (**f**) hepatic total cholesterol, and (**g**) malondialdehyde levels in mice. Data are presented as mean ± SD, based on eight mice per group (*n* = 8/group). Statistical analysis was performed using one-way ANOVA followed by Tukey’s post hoc test. Statistical significance is indicated as follows: ns, not significant, * *p* < 0.05, *** *p* < 0.001, and **** *p* < 0.0001.

The anti-obesity effects of FSPE were investigated using a high-fat diet (HFD)-induced obese mouse model. Mice fed the HFD exhibited significantly higher final body weight and greater overall weight gain compared to the ND group (Figure 2a,c). However, FSPE supplementation significantly reduced both body weight and weight gain in HFD-fed mice, while no significant difference in food intake was observed between the FSPE and HFD groups (Figure 2b). Notably, although liver weight, hepatic total cholesterol (TC), and malondialdehyde (MDA) levels did not exhibit significant differences among the groups (Figure 2d,f,g), hepatic triglyceride (TG) levels were markedly elevated in the HFD group and significantly reduced following FSPE supplementation (Figure 2e). These results suggested that FSPE primarily ameliorated hepatic triglyceride accumulation rather than altering cholesterol levels or oxidative stress markers in HFD-fed mice.

**Figure 3:**
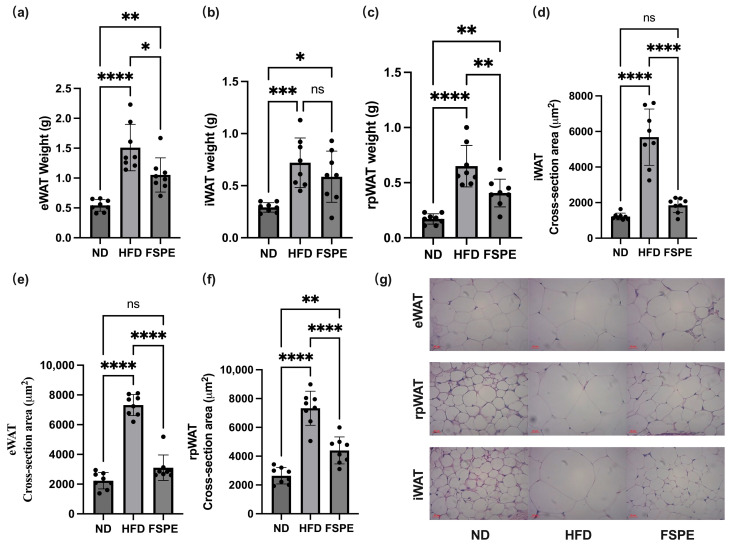
Effect of FSPE supplementation on adipose tissue mass and adipocyte size in HFD-fed mice. The weights of (**a**) epididymal white adipose tissue (eWAT), (**b**) inguinal white adipose tissue (iWAT), and (**c**) retroperitoneal (rpWAT) white adipose tissue in HFD-fed mice. The adipocyte area in (**d**) iWAT, (**e**) eWAT, and (**f**) rpWAT. (**g**) Representative H&E-stained adipose tissue sections. Scale bar: 20 μm. Data are presented as mean ± SD, based on eight mice per group (*n* = 8/group). Statistical analysis was performed using one-way ANOVA followed by Tukey’s post hoc test. Statistical significance is indicated as follows: ns, not significant,* *p* < 0.05, ** *p* < 0.01, *** *p* < 0.001, and **** *p* < 0.0001.

Analysis of fat accumulation revealed a marked increase in epididymal (eWAT) and retroperitoneal (rpWAT) white adipose tissue weights in HFD-fed mice, whereas FSPE supplementation significantly reduced fat accumulation in these regions (Figure 3a,c). Additionally, while inguinal white adipose tissue (iWAT) weight showed a decreasing trend in the FSPE group, the difference was not statistically significant (Figure 3b). These results suggest that FSPE suppresses visceral fat accumulation under HFD conditions.

Histological analysis of white adipose tissues using H&E staining revealed enlarged adipocytes in HFD-fed mice, which were significantly reduced in size following FSPE supplementation across all three adipose tissue sites (eWAT, rpWAT, and iWAT) (Figure 3d–g).

Collectively, these findings indicated that FSPE mitigated HFD-induced adiposity and hepatic lipid deposition, underscoring its potential therapeutic value for managing obesity and obesity-associated hepatic steatosis.

### 2.3. Effects of FSPE on Serum Biochemical Parameters in High-Fat Diet-Induced Obese Mice

To further evaluate the metabolic effects of FSPE, serum biochemical parameters were analyzed (Table 1). Compared to the ND group, HFD feeding significantly increased serum triglycerides (TG), total cholesterol (TC), and high-density lipoprotein cholesterol (HDL-C). FSPE supplementation markedly reduced TG and TC levels and slightly lowered HDL-C levels relative to the HFD group.

Markers of liver injury, including aspartate aminotransferase (AST) and alanine aminotransferase (ALT), were moderately elevated in the HFD group. FSPE administration led to slight reductions in both AST and ALT levels; however, these changes were not statistically significant.

Taken together, FSPE supplementation improved circulating lipid profiles and exerted a moderate effect on liver enzyme levels, further supporting its potential as a dietary intervention for HFD-induced metabolic disorders.

### 2.4. FSPE Increased SCFAs Production in High-Fat Diet-Induced Obese Mice

**Figure 4:**
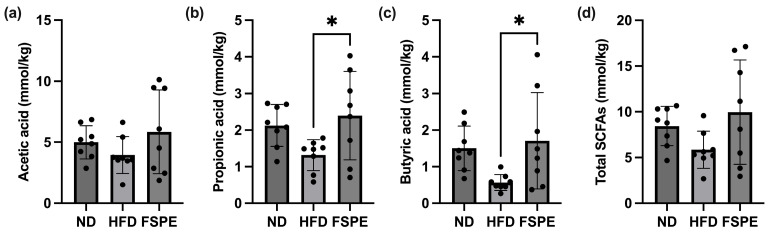
Effect of FSPE supplementation on fecal short-chain fatty acid (SCFA) levels in HFD-fed mice. The levels of (**a**) acetic acid, (**b**) propionic acid, (**c**) butyric acid, and (**d**) total fecal SCFA levels in mice feces. Data are presented as mean ± SD, based on eight mice per group (*n* = 8/group). Statistical analysis was performed using one-way ANOVA followed by Tukey’s post hoc test. Statistical significance is indicated as follows: * *p* < 0.05.

SCFAs are metabolically active compounds produced by the intestinal microbiota and are widely recognized as key indicators of intestinal health. To assess the impact of FSPE on SCFA production, the levels of acetic acid, propionic acid, and butyric acid in fecal samples were quantified using gas chromatography. No significant differences in acetic acid levels were observed among the experimental groups (Figure 4a). However, the FSPE group exhibited a significant increase in propionic acid levels compared to the HFD group (Figure 4b). Additionally, FSPE groups exhibited significantly elevated butyric acid levels relative to the HFD group (Figure 4c). Furthermore, while the FSPE group displayed higher total SCFAs levels than the HFD group, the difference was not statistically significant (Figure 4d). These findings suggest that FSPE may promote the production of propionic acid and butyric acid in the intestine. Nonetheless, the mechanisms underlying these changes remain to be elucidated.

### 2.5. FSPE Modulates the Expression of Genes Associated with Obesity and Inflammation in Adipose and Liver Tissues of HFD-Fed Mice

**Figure 5:**
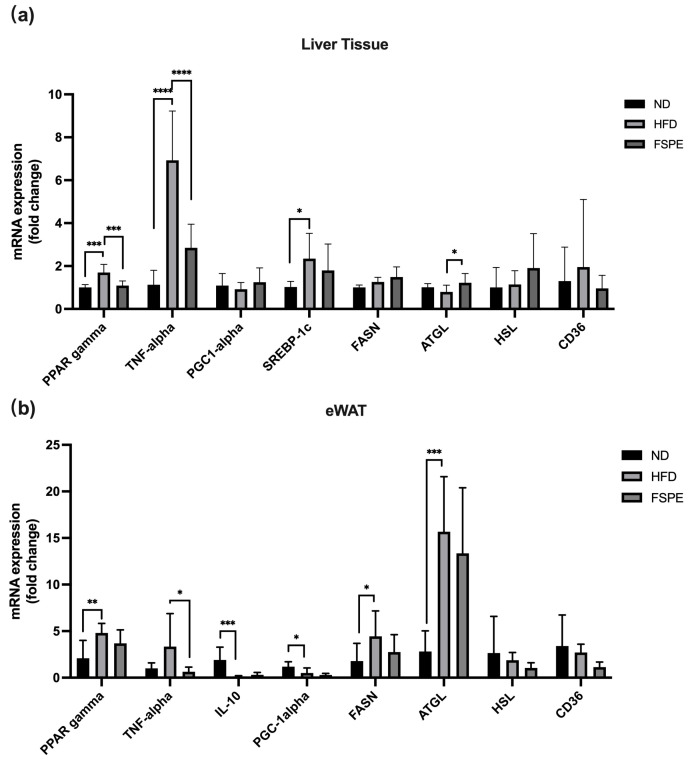
Effect of FSPE supplementation on inflammation-related gene expression in the liver and eWAT of HFD-fed mice. The gene expression profiles related to obesity, lipid metabolism, and inflammation were analyzed in (**a**) liver tissue and (**b**) eWAT samples from obese mice. Data were presented as mean ± SD, based on eight mice per group (*n* = 8/group). Statistical analysis was performed using one-way ANOVA followed by Tukey’s post hoc test. Statistical significance was indicated as follows: * *p* < 0.05, ** *p* < 0.01, *** *p* < 0.001, and **** *p* < 0.0001.

To elucidate the molecular mechanisms underlying the anti-obesity effects of FSPE, gene expression profiles related to obesity, lipid metabolism, and inflammation were analyzed in liver and eWAT samples. As shown in Figure 5a, HFD feeding significantly increased the hepatic expression of *PPARγ*, *TNF-α*, and *SREBP-1c* compared to the ND group. In contrast, FSPE supplementation significantly downregulated *PPARγ* and *TNF-α* while significantly upregulating *ATGL* expression compared to the HFD group. Although the ND group exhibited higher *PGC-1α* expression than the HFD group, the difference was not statistically significant. Similarly, while FSPE supplementation showed a trend toward increased *PGC-1α* levels, this elevation did not reach statistical significance. These results indicate that FSPE modulates *PPARγ*, *TNF-α,* and *ATGL*, thereby contributing to the mitigation of obesity and inflammation and the enhancement of lipolysis in the liver tissue.

In eWAT (Figure 5b), HFD feeding significantly upregulated *PPARγ*, *FASN*, and *ATGL*, while downregulating *IL-10* and *PGC-1α* compared to the ND group. However, FSPE supplementation did not exert significant regulatory effects on *PPARγ*, *IL-10*, or *PGC-1α*. Notably, FSPE markedly attenuated HFD-induced *TNF-α* upregulation. Furthermore, no significant differences in *HSL* and *CD36* expression were observed in either liver or eWAT across all groups. Collectively, these findings indicated that FSPE modulated key genes involved in obesity, lipid metabolism, and inflammation in a tissue-specific manner.

### 2.6. FSPE Modulates Serum Lipidomic Profiles

**Figure 6:**
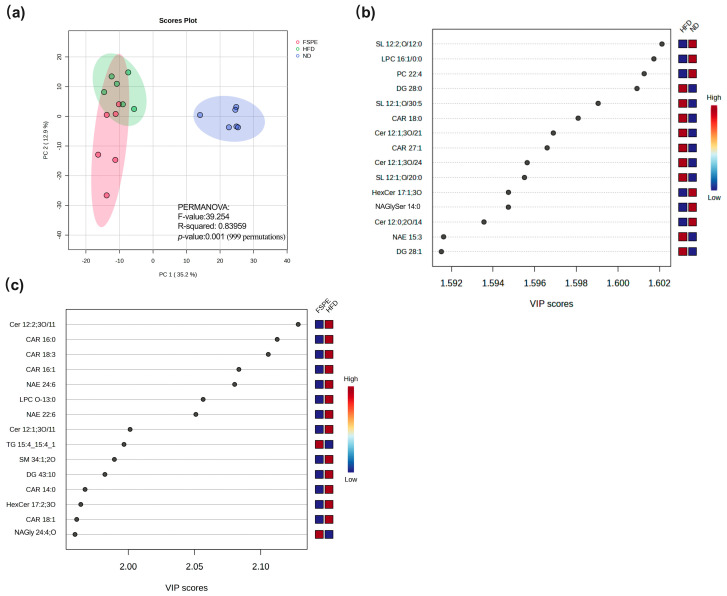
Effects of FSPE on serum lipidomic profiles in HFD-fed mice. (**a**) Principal component analysis (PCA) score plot; (**b**) Variable Importance in Projection (VIP) score analysis of top 15 discriminative lipid species (HFD vs. ND); (**c**) VIP score analysis of discriminative lipid species modulated by FSPE (FSPE vs. HFD). Data are based on *n* = 6 mice per group. The colored shaded regions represent the 95% confidence intervals for each treatment group.

To evaluate the impact of FSPE supplementation on systemic lipid metabolism, lipidomic profiling was performed on serum samples from the ND, HFD, and FSPE groups. Principal component analysis (PCA) revealed distinct separation among the three experimental groups (Figure 6a). The HFD group was clearly separated from the ND group along PC1 (35.2%), indicating substantial alterations in the serum lipidome induced by HFD feeding. Notably, the FSPE group showed partial clustering toward the ND group, suggesting that FSPE supplementation mitigated HFD-induced lipidomic disturbances.

Comparative lipidomic analysis between the ND and HFD groups identified multiple lipid species that were significantly elevated in the HFD group, as determined by Variable Importance in Projection (VIP) analysis (Figure 6b). Among the top-ranked lipids based on VIP scores, sphingolipids (SL 12:1;O/30:5, SL 12:1;O/20:0), ceramides (Cer 12:1;3O/21, Cer 12:1;3O/24), diacylglycerols (DG 28:0, DG 28:1), acylcarnitine (CAR 18:0, CAR 27:1), and N-acylethanolamines (NAE 15:3) were more abundant in the HFD group than in the ND group, indicating a broad spectrum of serum lipidomic alterations induced by HFD feeding.

**Figure 7:**
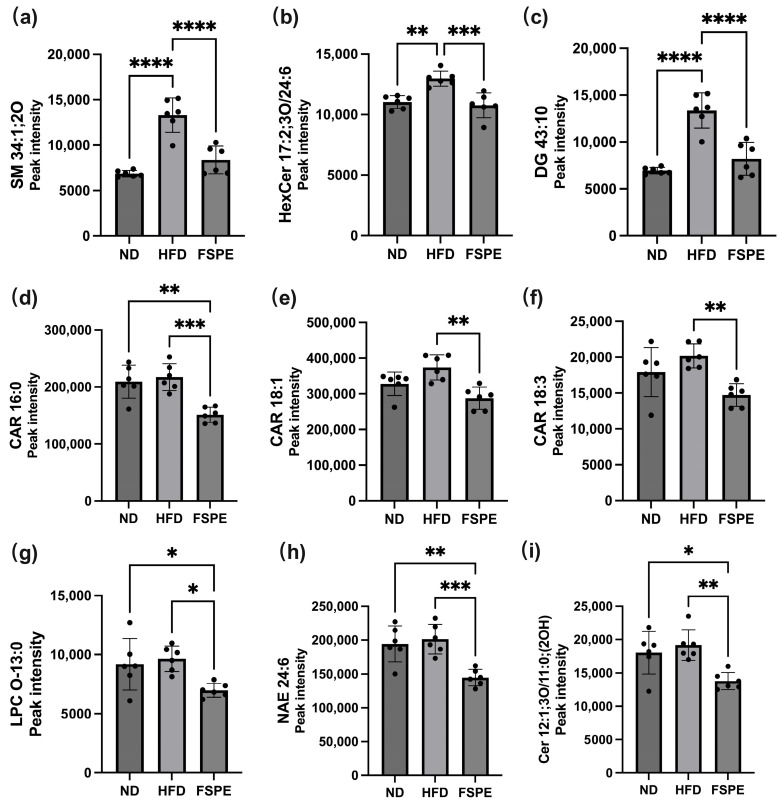
Effect of FSPE supplementation on HFD-induced lipid alterations in HFD-fed mice. The serum levels of (**a**) sphingomyelin (SM 34:1;2O), (**b**) hexosylceramide (HexCer 17:2;3O), (**c**) diacylglycerol (DG 43:10), (**d**–**f**) acylcarnitines (CAR 16:0, CAR 18:1, CAR 18:3), (**g**) lysophosphatidylcholine (LPC O-13:0), (**h**) N-acylethanolamine (NAE 24:6), and (**i**) ceramide [Cer 12:1;3O/11:0;(2OH)]. Data are presented as mean ± SD, based on six mice per group (*n* = 6/group). Statistical analysis was performed using one-way ANOVA followed by Tukey’s post hoc test. Statistical significance is indicated as follows: * *p* < 0.05, ** *p* < 0.01, *** *p* < 0.001, and **** *p* < 0.0001.

Comparison between the FSPE and HFD groups showed that FSPE supplementation reduced the levels of several lipid species (Figure 6c). Examination across all three groups (ND, HFD, and FSPE) revealed consistent trends indicative of FSPE-mediated modulation. Specifically, the levels of sphingomyelin (SM 34:1;2O), hexosylceramide (HexCer 17:2;3O), and diacylglycerol (DG 43:10) were elevated in the HFD group relative to ND, but were significantly reduced following FSPE supplementation (Figure 7a–c). Similarly, the levels of acylcarnitines (CAR 16:0, CAR 18:1, CAR 18:3), lysophosphatidylcholine (LPC O-13:0), N-acylethanolamines (NAE 24:6), and ceramides Cer 12:1;3O/11:0;(2OH) showed marked reductions in the FSPE group compared to the HFD group (Figure 7d–i).

Overall, these findings indicate that FSPE supplementation reverses key lipidomic alterations induced by HFD feeding, with elevated lipid species in HFD mice shifting toward ND levels following treatment.

### 2.7. Chemical Composition of FSPE

Metabolite analysis conducted to assess compositional changes before and after sweet potato fermentation revealed substantial increases in several bioactive compounds (Table 2). Notably, indolelactic acid levels rose approximately 114-fold following fermentation compared to unfermented sweet potatoes. Similarly, linoleic acid and its related derivatives showed remarkable increases, exceeding 100-fold. Analysis of polyphenols and flavonoids identified three key compounds—cedrelopsin, isorhamnetin, and scopoletin—as the most significantly elevated metabolites. Additionally, cedrelopsin and isorhamnetin increased by more than 20-fold, while scopoletine exhibited a six-fold increase.

## 3. Discussion

In recent years, there has been a growing interest in dietary strategies that incorporate functional foods and nutraceuticals as adjuncts to conventional treatments. In the present study, we investigated the effects of FSPE on obesity and related metabolic alterations in a HFD-induced obese mouse model. The results demonstrated that FSPE supplementation effectively reduced body weight gain, visceral fat accumulation, and hepatic triglyceride levels, while also modulating lipid metabolism, inflammatory gene expression, and circulating lipid profiles.

Lactic acid fermentation is a well-established pre-digestion process that enhances the release and transformation of bioactive compounds, improves bioavailability, and reduces sugar content [11]. By breaking down fiber and starch matrices, lowering pH, and facilitating the release of phenolic compounds, fermentation significantly enhances the functional properties of food [11,19]. Previous studies have shown that fermentation with *Lactobacillus plantarum* and *Lactobacillus casei* increases phenolic and flavonoid contents, thereby improving both antioxidant capacity and metabolic benefits [19].

In this study, fermentation using *L. amylovorus* OFMLa-73 and *L. brevis* OFMLb-143 significantly enhanced the production of bioactive compounds. It reduced reducing sugar content, lowered pH, and improved the stability of functional metabolites—all of which may contribute to metabolic health benefits [10,11]. Notably, indole lactic acid levels increased 114-fold following fermentation, likely contributing to pH reduction. This metabolite has been reported to promote the growth of beneficial bacteria, such as *Lactobacillus*, *Bifidobacterium*, and *Faecalibacterium*, while inhibiting potentially harmful genera, such as *Escherichia* and *Phascolarctobacterium* [20,21]. This microbial shift was associated with a significant increase in butyric acid levels observed in the FSPE group.

Additionally, linolenic acid and its derivatives increased more than 100-fold, consistent with previous reports showing that fermentation enhances the production of conjugated linolenic acid and its isomers. These compounds are associated with cardiovascular, anti-inflammatory, and metabolic health benefits [22,23]. Among polyphenols and flavonoids, cedrelopsin, isorhamnetin, and scopoletin showed the most pronounced enrichment, with more than 20-fold and 6-fold increases, respectively. These bioactive molecules have been linked to antioxidant, anti-inflammatory, and metabolic regulatory activities [24]. Furthermore, microbial fermentation facilitated flavonoid deglycosylation, potentially improving both bioavailability and functional efficacy [25]. Collectively, these findings highlight fermentation’s capacity to enhance the bioactive compound profile of sweet potato, with implications for metabolic regulation and modulation of the gut microbiota.

FSPE supplementation significantly reduced adipose tissue accumulation, decreased hepatic lipid droplet formation, and attenuated triglyceride accumulation in HFD-fed mice, suggesting its potential to mitigate obesity-related complications. These effects are likely due to the synergistic actions of fermentation-derived bioactive compounds, including indole lactic acid, conjugated linolenic acid, cedrelopsin, isorhamnetin, and scopoletin, which may collectively improve metabolic outcomes. Interestingly, FSPE reduced body weight and adiposity without affecting food intake, suggesting increased energy expenditure. Although we did not measure thermogenic markers such as UCP1 in this study, previous reports indicate that metabolites, particularly indole derivatives, can modulate energy metabolism [26]. It is plausible that FSPE exerts its anti-obesity effects, at least in part, by enhancing thermogenesis, a mechanism warranting further investigation. Additionally, SCFAs play a key role in mediating interactions between diet, gut microbiota, and host health. SCFAs are bioactive fermentation products that enhance gut barrier function and support metabolic homeostasis [27].

It is well-established that an HFD promotes obesity and adipocyte hypertrophy and disrupts the gut microbiota, leading to reduced microbial diversity and diminished SCFA production [28,29]. Phenolic-rich extracts have been shown to enhance SCFA production by increasing the abundance of butyrate-producing bacteria, including *Ruminococcus* and *Akkermansia* [30]. Similarly, lychee phenolic extracts significantly elevate the levels of acetic acid, propionic acid, and valeric acid during in vitro fecal fermentation [31], suggesting that phenolic compounds and flavonoids play a crucial role in stimulating SCFA production. Taken together, physiological measurements, histological evaluation, and SCFA profiling consistently underscore the anti-obesity potential of FSPE, reinforcing its promise as a functional dietary intervention for improving metabolic health.

To further assess the metabolic effects of FSPE, the relative expression of key metabolic and inflammatory genes was analyzed in the liver and eWAT. *PPARγ* plays a central role in adipocyte differentiation, lipid metabolism, and inflammation, and is activated by natural ligands such as fatty acids and prostaglandins [32]. In this study, *PPARγ* expression was elevated in the livers of obese mice but was significantly reduced following FSPE supplementation, suggesting an improvement in metabolic function. Given its association with hepatic steatosis and insulin sensitivity, the downregulation of *PPARγ* in FSPE-treated mice may be mediated by SCFA-induced promotion of fatty acid oxidation and reduction in triglyceride accumulation [33]. In addition, our results showing elevated *ATGL* mRNA in eWAT of obese mice were consistent with previous findings [34]. *ATGL* expression in WAT may respond to insulin resistance and increased lipolysis demands [34]. On the other hand, FSPE upregulated hepatic *ATGL* expression in HFD-fed mice. Being a critical enzyme responsible for lipolysis, *ATGL* promotes the breakdown of stored triglycerides [35]. Consistent with previous findings [36], our results suggested that the FSPE supplementation modulated hepatic fatty acid metabolism more effectively than cholesterol homeostasis. This difference may be attributed to the rapid modulation of lipogenesis, whereas promoting cholesterol excretion may require a more extended intervention period.

Similarly, *TNF-α*, secreted by Kupffer cells in the liver and by infiltrating immune cells in adipose tissue, plays a key role in obesity-induced inflammation. Elevated *TNF-α* levels activate the NF-κB pathway, contributing to the progression of MASLD [37,38]. In this study, *TNF-α* expression was significantly elevated in the liver and eWAT of obese mice, whereas FSPE supplementation effectively reduced *TNF-α* levels. This anti-inflammatory effect is likely mediated by prebiotic compounds such as polyphenols, flavonoids, and anthocyanins, which enhance SCFA production, thereby promoting homeostasis in the gut–liver axis [39]. Similar effects have been observed with flavonoid-rich extracts, which attenuate NF-κB activity and inhibit pro-inflammatory signaling [40,41].

Although qPCR analysis provided valuable insights into gene regulation, further research is needed to elucidate the complex interactions among HFD-induced MASLD, gut microbiota, and metabolic pathways. Future investigations should elucidate the mechanisms by which FSPE and its bioactive constituents modulate MASLD progression, with particular emphasis on microbiota–metabolite interactions.

The lipid alterations observed in this study are consistent with previous reports linking HFD consumption to elevated levels of sphingomyelin, hexosylceramide, and diacylglycerol. Both sphingomyelin and ceramide are recognized as bioactive lipids that accumulate in response to HFD and contribute to insulin resistance, chronic inflammation, and aberrant lipid storage [42]. Elevated serum ceramide species have been shown to mediate the metabolic effects of excessive saturated fatty acid intake and inflammatory cytokines such as *TNF-α*, ultimately promoting insulin resistance [43,44]. Similarly, elevated hexosylceramide levels in obesity have been associated with increased adipocyte hexosylceramide synthase expression, potentially contributing to impaired insulin signaling [45]. Acylcarnitines, particularly medium- and long-chain species, are frequently elevated under conditions of impaired fatty acid oxidation and are considered markers of metabolic inflexibility [46]. Additionally, imbalances in N-acylethanolamines (NAEs)—including altered concentrations and ratios—have been reported in obesity, indicating disrupted lipid-derived signaling [47].

Importantly, our data align with previous studies showing that phytochemical-rich interventions—including polyphenols, flavonoids, and fermentation-derived plant metabolites—can mitigate HFD-induced lipidomic disruptions. For example, fermented mulberry leaves have been shown to reduce backfat thickness and downregulate triglyceride accumulation in pig adipose tissue [48]. Similarly, lipidomics analysis of fermented tomato products revealed significant modulation of glycerophospholipids, sphingolipids, unsaturated fatty acids, and amino acid profiles [49]. It is worth noting that while unfermented purple sweet potato has been reported to exhibit anti-obesity effects [50], this study focused specifically on the therapeutic potential of the fermented extract (FSPE). As evidenced by our metabolite analysis (Table 2), fermentation dramatically altered the chemical composition, increasing the levels of key bioactive metabolites—such as indolelactic acid—by over 100-fold. Given this fundamental shift in the phytochemical profile, FSPE was investigated as a distinct functional ingredient. However, a comparison between fermented and unfermented extracts would further indicate the specific contributions of fermentation-derived metabolites. While the extracted metabolites were identified as the primary active components responsible for the observed systemic effects, future research investigating the structural properties of the fermented purple sweet potato matrix would provide a more comprehensive understanding of the biotransformation process. These remain the limitation of the present study and should be addressed in future investigations. Taken together, the lipidomic findings in this study are well supported by previous nutrition-based intervention research, reinforcing the lipid-modulatory potential of FSPE in the context of metabolic stress.

## 4. Materials and Methods

### 4.1. Fermentation of Sweet Potato Samples

The purple sweet potato variety Tainong No. 73 was sourced from Yifa Commercial Farm (Yunlin, Taiwan). Two LAB strains—*Lactobacillus amylovorus* OFMLa-73 and *Levilactobacillus brevis* OFMLb-143—were isolated from pig feces and fermented sauerkraut, respectively, and cultured in MRS broth (Neogen Corporation, Lansing, MI, USA). The fermentation process followed a previously described method [12], with modifications. Steamed sweet potato was mixed with the LAB strains in 0.85% NaCl solution at a solid-to-liquid ratio of 1:1, and incubated at 37 °C for 48 h. After fermentation, the samples were frozen at −80 °C, dried using a floor-type vacuum freeze dryer for 48 h, and stored at −80 °C until further use.

### 4.2. Extraction of Fermented Purple Sweet Potato

Fermented sweet potato was extracted using two methods: hot water extraction and ethanol extraction. For hot water extraction, 30 g of freeze-dried fermented purple sweet potato was mixed with 170 mL of deionized water and stirred at 600 rpm for 10 min. The mixture was then extracted in a water bath at 80 °C for 2 h; this process was repeated three times. After extraction, the mixture was centrifuged at 7800× *g* for 10 min, and the supernatant was collected and frozen at −80 °C. The following day, the extract was dried using a vacuum freeze dryer (FD10/-80, Firstek Scientific Co., Ltd., New Taipei City, Taiwan) and stored at −80 °C for subsequent experiments.

Ethanol extraction was performed according to a previously described method [12], with modifications. Briefly, freeze-dried fermented sweet potato was extracted with 70% ethanol at a 1:10 volume ratio for 1 h. The mixture was centrifuged at 7800× *g* and filtered through filter paper to collect the supernatant; this process was repeated three times. The collected supernatants were combined and concentrated under reduced pressure using a rotary evaporator (N-1000, EYELA, Tokyo, Japan). Subsequently, the extract was frozen at −80 °C, dried using a vacuum freeze dryer (FD10/-80, Firstek Scientific Co., Ltd., New Taipei City, Taiwan), and stored at −80 °C until use.

### 4.3. Total Phenolic Content

Total phenolic content was analyzed using a previously reported method [12], with minor modifications. Briefly, 100 μL of the fermented sweet potato extract (FSPE) was mixed with 50 μL of Folin–Ciocalteu reagent (Sigma-Aldrich, St. Louis, MO, USA) and 300 μL of 2% sodium carbonate (Yakuri Pure Chemicals, Uji, Japan). The mixture was incubated at 25 °C for 15 min, after which 1 mL of distilled water was added. Absorbance was measured at 725 nm using a microplate reader (Thermo Fisher Scientific, St. Louis, MO, USA). Results were expressed as milligrams of gallic acid equivalents (mg GAE/g DW) per gram of dry weight, based on a standard curve prepared using gallic acid (Macklin, Shanghai, China).

### 4.4. Total Flavonoid Content

Total flavonoid content was analyzed as described in a previous study [51], with minor modifications. FSPE or quercetin standard (Sigma-Aldrich, St. Louis, MO, USA) was dissolved in 1 mL of methanol, then mixed with 4 mL of 60% ethanol and 0.3 mL of 5% sodium nitrite (Duksan, Ansan, Republic of Korea). After reaction, 10% aluminum trichloride (Thermo Fisher Scientific, St. Louis, MO, USA) was added. After an additional 6 min of incubation, the reaction was terminated by adding 4% NaOH (Honeywell International, Frankfurt, Germany), and the reaction mixture was diluted to 10 mL with 60% ethanol. After 15 min, the absorbance was measured at 510 nm using a microplate reader (Synergy H1, Agilent Technologies, Santa Clara, CA, USA). A standard curve was generated using quercetin, and the total flavonoid content was expressed as milligrams of quercetin equivalents per gram of dry weight (mg QCE/g DW) of the extract.

### 4.5. Total Anthocyanin Content

Total anthocyanin content was determined following a previously reported method [12], with modifications. Briefly, 1 mL of FSPE was mixed with either 500 μL of 0.025 M hydrochloric acid–potassium chloride buffer (pH 1.0; Thermo Fisher Scientific, St. Louis, MO, USA) or 500 μL of 0.4 M sodium acetate buffer (pH 4.5; Sigma-Aldrich, St. Louis, MO, USA). The mixtures were incubated at 25 °C for 15 min. Absorbance was measured at 530 nm and 700 nm using a microplate reader. The total anthocyanin content was expressed as milligrams of anthocyanin-3-glucoside (Sigma-Aldrich, St. Louis, MO, USA) equivalents per gram of dry weight (mg/g DW) of the extract.

### 4.6. Reducing Sugar Content

Reducing sugar content was analyzed following previously reported methods [52,53], with modifications. A standard curve was prepared using glucose (Neogen Corporation, Lansing, MI, USA) at concentrations ranging from 0 to 1 mg/mL. A 0.5 mL aliquot of the sample solution was mixed with 1.5 mL of 3,5-dinitrosalicylic acid reagent and heated at 100 °C for 10 min. After cooling to 25 °C in an ice bath, 8 mL of deionized water was added to the sample, and its absorbance was measured at 540 nm using a microplate reader. Reducing sugar content was expressed as milligrams of glucose per gram of dry weight (mg GLU/g DW) of the extract.

### 4.7. Animal Handling and Treatment

Six-week-old male C57BL/6 mice were obtained from the National Center for Biomodels (Hsinchu, Taiwan) and housed at the Terrestrial Animal Experiment Center of National Taiwan Ocean University. All procedures were approved by the Institutional Animal Care and Use Committee of National Taiwan Ocean University (Approval No. 111021). Environmental conditions were maintained at 23 ± 3 °C, 40–60% humidity, and a 12 h light/dark cycle. Mice were provided chow diets and deionized water ad libitum.

After a two-week acclimatization period, mice were fed either a 10% kcal fat normal diet (D12450J, Research Diets) or a 60% kcal high-fat diet (D12492, Research Diets) for 10 weeks. Mice were randomly assigned to three groups (*n* = 8/group): normal diet (ND), high-fat diet (HFD), and high-fat diet supplemented with FSPE (FSPE; 1000 mg/kg BW/day/200 μL). The animal dosage of FSPE was based on a similar study Shin et al. 2013 [54], which demonstrated effective metabolic modulation at this concentration without adverse effects. After 10 weeks, mice were sacrificed, and orbital blood and fecal samples were collected. Liver and white adipose tissues were harvested. A portion of the tissues was immediately fixed in 10% formalin for histological analysis, while the remaining tissues were rapidly frozen at −80 °C for subsequent biochemical experiments.

### 4.8. Liver Triglycerides Test

Liver triglyceride levels were measured using a colorimetric assay kit (Cayman Chemical, Ann Arbor, MI, USA) according to the manufacturer’s instructions. Briefly, a liver sample from each experimental group was homogenized in NP40 substitute reagent. The prepared samples and standards were individually mixed with the triglyceride enzyme reagent and incubated at 37 °C for 30 min. Absorbance was measured at 540 nm using a microplate reader.

### 4.9. Liver Total Cholesterol Test

Liver total cholesterol levels were determined using a colorimetric assay kit (Randox Laboratories, Crumlin, UK) according to the manufacturer’s instructions. Briefly, approximately 10 mg of liver tissue was homogenized in 200 μL of chloroform/isopropanol/Triton X-100 solution (7:11:0.1, *v*/*v*/*v*) [55]. Samples were first manually ground using a pestle, then ceramic beads were added, and the mixture was subjected to high-speed shaking at 8000 rpm for 2 cycles of 30 s, with a 15 s interval between cycles. The homogenates were then centrifuged at 12,000 rpm for 10 min, and the supernatants were transferred to new tubes. Organic solvents were evaporated by air-drying at 50 °C to remove chloroform. The dried lipid extracts were subsequently resuspended in 200 μL of isopropanol and sonicated until fully dissolved. The total cholesterol content was finally quantified using the manufacturer’s enzymatic colorimetric method, and absorbance was measured at 500 nm using a microplate reader.

### 4.10. Measurement of MDA in Liver Homogenate

MDA levels were determined using the thiobarbituric acid reactive substances (TBARS) assay [56]. Liver tissues were weighed and homogenized in ice-cold 1.15% potassium chloride solution at a 1:9 (*w*/*v*) ratio. An aliquot of the homogenate was mixed with sodium dodecyl sulfate, acetic acid (pH 3.5), and thiobarbituric acid, and the reaction mixture was heated at 95 °C for 60 min. After cooling, the reaction products were extracted with n-butanol and centrifuged to collect the organic phase. The absorbance was measured at 532 nm using a microplate reader. MDA concentrations were calculated from a standard curve prepared with 1,1,3,3-tetramethoxypropane and expressed as nmol MDA per gram of wet tissue.

### 4.11. Pathological Tissue Analysis

Post-sacrifice, tissues were fixed in 10% formaldehyde solution for 24 h. Histological sectioning and hematoxylin and eosin (H&E) staining were performed by the Center of Excellence for the Oceans, National Taiwan Ocean University. For adipocyte analysis, 60 adipocytes were randomly selected from each sample, and their cell areas were calculated.

### 4.12. Real-Time Quantitative Polymerase Chain Reaction (qPCR)

Total RNA was extracted using the Total RNA Extraction Kit (Viogene, New Taipei, Taiwan). cDNA synthesis was performed using the High-Capacity cDNA Reverse Transcription Kit (Thermo Fisher Scientific, St. Louis, MO, USA). Real-time qPCR was performed using SYBR Green dye (HighQu, Kraichtal, Germany) and a real-time nucleic acid analyzer (Thermo Fisher Scientific, St. Louis, MO, USA). Primer sequences are listed in Table S1.

### 4.13. Fecal Short-Chain Fatty Acid (SCFA) Analysis

SCFA levels were analyzed using a previously reported method [29], with minor modifications. SCFAs were extracted from a 50 mg sample mixed with internal standard (2-ethylbutyric acid) (Sigma-Aldrich, St. Louis, MO, USA) and supersaturated saline. The mixture was acidified with 10% sulfuric acid, vortex-extracted with ether (Echo Chemical, Toufen, Taiwan), and centrifuged. The supernatant was dried using anhydrous sodium sulfate to remove moisture, centrifuged again, filtered (0.22 μm PVDF), and subjected to GC-FID analysis. Analytical-grade acetic acid, propionic acid, and butyric acid were used as mixed standards, with 2-ethylbutyric acid as the internal standard to generate a calibration curve. SCFA concentrations (mmol/kg) were calculated based on retention times and the standard curve.

### 4.14. Lipidomics Analysis

Lipidomic profiling was performed by the National Microbiome Core Facility, Taiwan. Briefly, mouse serum samples were precipitated with methanol, followed by centrifugation at 10,000× *g* to pellet the precipitated proteins. The resulting supernatant was collected and filtered through a 0.22 µm regenerated cellulose membrane (RC-4, Sartorius, Göttingen, Germany), then stored at −20 °C until further analysis. Lipidomic profiling was conducted using an Agilent 1290 ultra-high-performance liquid chromatography (UHPLC) system coupled with a Bruker maXis ultra-high-resolution time-of-flight (UHR-TOF) mass spectrometer (Bruker Daltonics, Bremen, Germany). Lipid separation was achieved using an Agilent InfinityLab Poroshell HPH-C18 column (2.1 × 100 mm, 1.9 μm; Agilent Technologies, Santa Clara, CA, USA), with electrospray ionization (ESI) performed in positive mode. Each serum sample was analyzed in triplicate.

Raw data from the maXis UHR-TOF were processed using MS-DIAL version 4.60. Peak detection and alignment were performed with a minimum peak height threshold of 10,000 and a retention time tolerance of 0.2 min; all other parameters were set to default values as recommended by the software. Unknown lipid features were identified using the MS-DIAL metabolomics MSP spectral kit from MS-DIAL. Final lipid profiles were processed and analyzed using MetaboAnalyst 6.0.

### 4.15. Analysis of Chemical Compounds from Fermented Sweet Potato Extract (FSPE)

The chemical compounds in FSPE were analyzed using an Agilent LC/Q-TOF 6546 system (Agilent Technologies, Santa Clara, CA, USA) equipped with an Acquity UPLC BEH C18 UPLC column (2.1 × 100 mm, 1.7 μm particle size) (Waters Corporation, Milford, MA, USA). The mobile phases consisted of ultrapure water (A) and LC-MS grade acetonitrile (B), both containing 0.1% formic acid. The system was initially equilibrated with 5% solvent B for 2 min, followed by a linear gradient to 100% B over 17 min. This was maintained for 3 min, then rapidly returned to 5% B over 0.1 min, with a final re-equilibration period of 2.9 min. Mass spectrometry was conducted in positive ion mode using a Dual Agilent Jet Stream ESI source (Agilent Technologies, Santa Clara, CA, USA). Key operating parameters included a sheath gas temperature of 350 °C, a flow rate of 11.0 L/min, a fragmentor voltage of 140 V, a capillary voltage of 3.5 kV, and a nebulizer pressure of 40 psig. The drying gas was set to 320 °C with a flow rate of 12 L/min. Collision energies were applied at 20 and 40 eV, and the ion spray voltage was maintained at 0 V. MS2 acquisition was limited to a maximum of five precursors per cycle, with dynamic exclusion applied after three spectra within 0.2 min. MS1 scans were recorded over an *m*/*z* range of 100–1700, while MS2 scans covered *m*/*z* 20–1700, at a scan speed of 8 spectra/s. The absolute intensity threshold was set at 500 counts, and mass tolerance was maintained at 20 ppm.

### 4.16. Statistical Analysis

All data and graphical outputs were analyzed using GraphPad Prism 9.0 software. Differences between experimental groups were assessed using one-way analysis of variance (ANOVA). Post hoc comparisons were performed using Tukey’s and Dunnett’s tests to determine statistical significance. Results are presented as mean ± standard deviation (SD), and statistical significance was set at *p* < 0.05.

## 5. Conclusions

In conclusion, this study demonstrated that FSPE produced using *L. amylovorus* OFMLa-73 and *L. brevis* OFMLb-143 significantly reduced lipid accumulation and hepatic triglyceride levels in HFD-induced obesity mice. FSPE notably enhanced SCFA production, which may contribute to suppressed lipogenesis and improved lipid metabolism. Furthermore, lipidomic analysis revealed that FSPE modulated serum levels of acylcarnitines and sphingolipids, indicating regulatory effects on lipid metabolism. Fermentation by these specific probiotic strains enriched purple sweet potato with several bioactive metabolites, including indolelactic acid and polyphenolic compounds, which may contribute to the observed anti-obesity effects by modulating key metabolic pathways. Therefore, FSPE presents a promising dietary intervention for managing obesity and improving lipid homeostasis. Nevertheless, further research is needed to elucidate the mechanisms underlying FSPE’s bioactivity and assess its efficacy in human clinical trials.

## Figures and Tables

**Table 1:** Effects of FSPE supplementation on serum biochemical parameters in HFD-fed mice.

Item	ND	HFD	FSPE
AST (U/L)	46.26 ± 11.88 ^a^	61.20 ± 18.89 ^a^	58.76 ± 23.05 ^a^
ALT (U/L)	9.64 ± 1.31 ^b^	28.98 ± 23.75 ^a^	16.94 ± 5.24 ^a^
TC (mg/dL)	68.20 ± 6.79 ^c^	101.81 ± 8.58 ^a^	91.10 ± 5.10 ^b^
TG (mg/dL)	7.10 ± 2.30 ^c^	26.29 ± 6.28 ^a^	16.91 ± 5.75 ^b^
HDL-C (mg/dL)	53.26 ± 5.73 ^c^	81.45 ± 6.36 ^a^	73.78 ± 4.01 ^b^
LDL-C (mg/dL)	11.33 ± 1.50 ^a^	10.56 ± 2.06 ^a^	9.49 ± 1.49 ^a^

Values with different letters in the same row indicated statistical significance (*p* < 0.05, mean ± SD, *n* = 8).

**Table 2:** Fold change values of up-regulated chemical compounds in FSPE.

Compounds	*m*/*z*	FSPE (Peak int.)	NNF ^1^ (Peak int.)	Fold Change (FSPE/NNF)
9(10)-EpOME	279.232	9.05 × 10^6^	7.55 × 10^4^	119.868
Indolelactic acid	206.081	3.70 × 10^6^	3.00 × 10^4^	114.907
Conjugated linoleic Acid (10E,12Z)	263.237	7.40 × 10^6^	6.50 × 10^4^	113.846
Linoleic acid	281.248	5.05 × 10^6^	4.90 × 10^4^	103.061
Cedrelopsin	261.113	1.03 × 10^6^	4.35 × 10^4^	23.563
Isorhamnetin (Quercetin 3′-methyl ether)	317.066	6.25 × 10^5^	3.00 × 10^4^	20.833
Ethyl pyroglutamate	158.081	1.05 × 10^6^	1.60 × 10^5^	6.531
Scopoletin	193.05	4.10 × 10^7^	6.85 × 10^6^	5.985
pimelic acid	183.05	2.60 × 10^5^	6.00 × 10^4^	4.333
pyridoxal	150.055	6.90 × 10^5^	2.75 × 10^5^	2.509
Oxidised Phytochelatin 2	538.128	1.95 × 10^5^	8.65 × 10^4^	2.254
13S-Hydroxy-9Z,11E,15Z-octadecatrienoic acid	277.217	1.55 × 10^5^	6.95 × 10^4^	2.23
3-methylquercetin	317.066	6.85 × 10^5^	3.15 × 10^5^	2.175

^1^ NNF: Extract of non-fermented purple sweet potato.

## Data Availability

Dataset available on request from the authors.

## Supplementary Materials

The following supporting information can be downloaded at: https://www.mdpi.com/article/10.3390/ijms27031489/s1. Refs. [57,58,59,60,61,62] are cited in Supplementary Materials file.

## Abbreviations

The following abbreviations are used in this manuscript:

MASLD	Metabolic dysfunction-associated steatotic liver disease
LAB	Lactic acid bacteria
TPC	Total phenolic content
TFC	Total flavonoid content
TAC	Total anthocyanin content
SCFA	Short-chain fatty acid
HFD	High-fat diet
WAT	White adipose tissue
PCA	Principal component analysis
VIP	Variable Importance in Projection
*PPARγ*	Peroxisome proliferator-activated receptor gamma
*TNF-α*	Tumor necrosis factor-alpha
*SREBP-1c*	Sterol regulatory element-binding protein 1c
*ATGL*	Adipose triglyceride lipase
*PGC-1α*	Peroxisome proliferator-activated receptor gamma coactivator 1-alpha
