# Supplementary material for: Targeted Lipidomics and Lipid Metabolism Elucidate Anti-Obesity Effects of Lactic Acid Bacteria-Fermented Purple Sweet Potato Tainung No. 73 Extract in Obese Mice
Source: Int J Mol Sci. 2026 Feb 3;27(3):1489. doi: 10.3390/ijms27031489 (PMC12898302; doi:10.3390/ijms27031489)
Supplement: Supplementary file 1 [file ijms-27-01489-s001.zip › ijms-4081454-Supplementary information.pdf]

Targeted Lipidomics and Lipid Metabolism Elucidate Anti-Obesity Effects of Lactic Acid Bacteria-Fermented Purple Sweet Potato Tainung No. 73 Extract in Obese Mice

Hsien-Yi Yang[1#], Chien-Hsun Huang[2#], Shang-Tse Ho[3], Hsin-Hui Su[1], Yen-Po Chen[4,5], Yung-Tsung Chen[1*]

[1] Department of Food Science, National Taiwan Ocean University, Keelung City 202301, Taiwan.
[2] Bioresource Collection and Research Center (BCRC), Food Industry Research and Development Institute, Hsinchu 30062, Taiwan.
[3] Department of Wood Based Materials and Design, College of Agriculture, National Chiayi University, Chiayi. 600, Taiwan.
[4] Department of Animal Science, National Chung Hsing University, Taichung City 40227, Taiwan.
[5] The iEGG and Animal Biotechnology Research Center, National Chung Hsing University, Taichung City 40227, Taiwan.

[#]Hsien-Yi Yang and Chien-Hsun Huang contributed equally to this work.

***Corresponding author:**

Dr. Yung-Tsung Chen

Department of Food Science, National Taiwan Ocean University.

No. 2 Beining Road, Zhongzheng District, Keelung City 202301, Taiwan.

Email Address: ianchen619@mail.ntou.edu.tw

Table S1. Primers employed in quantitative real-time PCR.

Gene	Forward primer (5’ →3’)	Reverse primer(3’→5’)	Reference
GAPDH	ACTCCACTCACGGCAAATTC	TCTCCATGGTGGTGAAGACA	[57]
PPAR-γ	GGAAGACCACTCGCATTCCTT	GTAATCAGCAACCATTGGGTCA	[12]
TNF-α	TGGTTTGGGAGAGAGTTGCA	CCTGTATATACGAAGCTTATT	[60]
IL-10	GCTGGACAACATACTGCTAACC	ATTTCCGATAAGGCTTGGCAA	[61]
SREBP-1C	GATGCCTGCCTACACATAGAAG	GGAGCCTCGATACTTGCTGGC	[60]
PGC-1α	TCTGAGTCTGTATGGAGTGACAT	CCAAGTCGTTCACATCTAGTTCA	[62]
FASN	GATTCAGGGAGTGGATATTG	CATTCAGAATTCGTGGCATAG	[58]
ATGL	GTCCTTCACCATCCGCTTGTT	CTCTTGGCCCTCATCACCAG	[59]
HSL	GCAAGATCAAAGCCTCAGCG	GCCATATTGTCTTCTGCGAGTGT	[59]
CD36	AGATGACGTGGCAAAGAACAG	CCTTGGCTAGATAACGAACTCTG	[59]

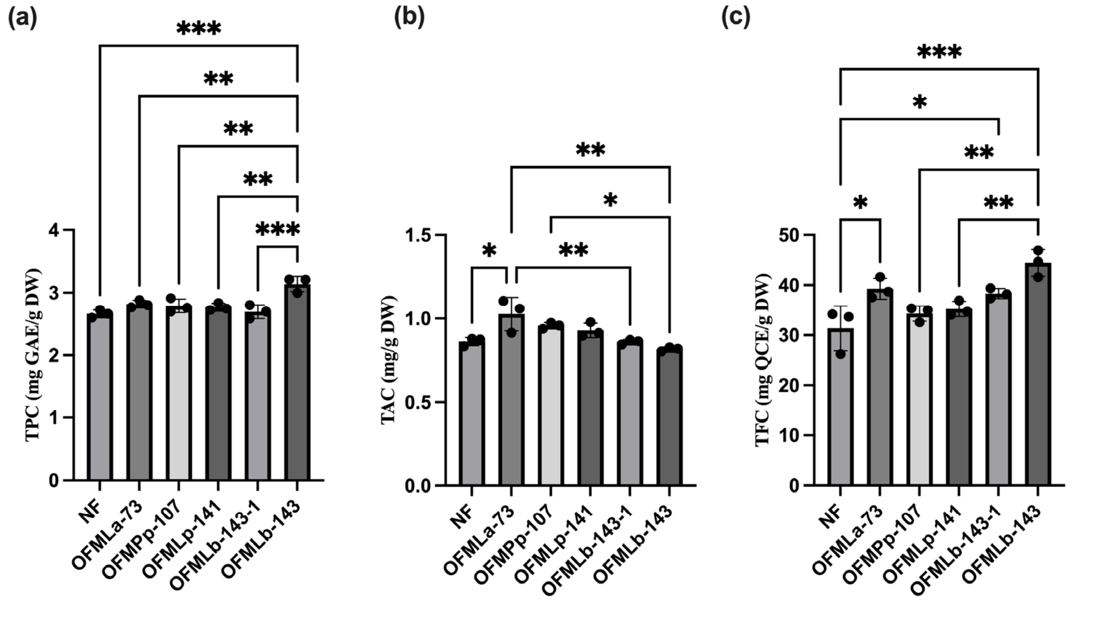

Figure S1. Screening of lactic acid bacteria strains for purple sweet potato fermentation. The effects of five different LAB strains on the levels of (a) total phenolic content, (b) total anthocyanin content, and (c) total flavonoid content in water extract of fermented sweet potato. Data are presented as mean ± SD. (*n*=3 / group)
